# Two Engineered OBPs with opposite temperature-dependent affinities towards 1-aminoanthracene

**DOI:** 10.1038/s41598-018-33085-8

**Published:** 2018-10-04

**Authors:** Filipa Gonçalves, Tarsila G. Castro, Nuno G. Azoia, Artur Ribeiro, Carla Silva, Artur Cavaco-Paulo

**Affiliations:** Centre of Biological Engineering, University of Minho, Campus de Gualtar, 4710-057 Braga, Portugal

## Abstract

Engineered odorant-binding proteins (OBPs) display tunable binding affinities triggered by temperature alterations. We designed and produced two engineered proteins based on OBP-I sequence: truncated OBP (tOBP) and OBP::GQ_20_::SP-DS3. The binding affinity of 1-aminoanthracene (1-AMA) to these proteins revealed that tOBP presents higher affinity at 25 °C (kd = 0.45 μM) than at 37 °C (kd = 1.72 μM). OBP::GQ_20_::SP-DS3 showed an opposite behavior, revealing higher affinity at 37 °C (kd = 0.58 μM) than at 25 °C (kd = 1.17 μM). We set-up a system containing both proteins to evaluate their temperature-dependent binding. Our data proved the 1-AMA differential and reversible affinity towards OBPs, triggered by temperature changes. The variations of the binding pocket size with temperature, confirmed by molecular modelling studies, were determinant for the differential binding of the engineered OBPs. Herein we described for the first time a competitive temperature-dependent mechanism for this class of proteins.

## Introduction

Proteins have crucial roles as components of stimulus-responsive systems from molecular to macroscopic level. The ability of proteins to change their conformation and physical properties in response to differentiate stimuli is ubiquitous in nature^1^. Exploiting the stimulus-responsive nature of proteins, novel materials have been designed to respond to different external stimuli such as light, pH, ion and metal concentrations, temperature, electrical potential, redox state or the presence of specific biomolecules^2–7^. This response involves mainly protein conformational changes which can be tuned for the development of biological applications, including biomaterials, nanodevices, biosensors, tissue engineering and drug or gene delivery^1,8–11^.

We explore for the first time odorant binding proteins (OBPs) as a new class of thermo-responsive proteins. OBP-I is an odorant-binding protein purified from the nasal mucosa of pig with 157 amino acids with known structure (PDB code 1A3Y)^12^. The protein has 8-stranded β-barrel flanked by an α-helix at the C-terminal end of the polypeptide chain^12,13^. OBP-I has the capacity to bind different ligands with varied functional groups and diverse carbon backbones such as terpenoids, aromatic compounds, aliphatic molecules and aldehydes^14^. Several biotechnological applications, including food safety^15^, disease diagnostics^16^ and environmental monitoring^17^ have been developed based on the reversible binding capability of OBP-I^18^, on its high broad spectra of detection, thermal stability, sensitivity, resistance to organic solvents and pH variation. OBPs have been studied as biosensors (i) for environmental monitoring and detection of dangerous substances^19^; (ii) fabrication of cartridges for removing the herbicide atrazine; and cleaning of waste waters^20^; (iii) for detection of food contaminants^15,21^; (iv) for the detection of explosive compounds^22^; and (v) reduction of unpleasant odors and controlled release of fragrances^23^.

**Figure 1:**
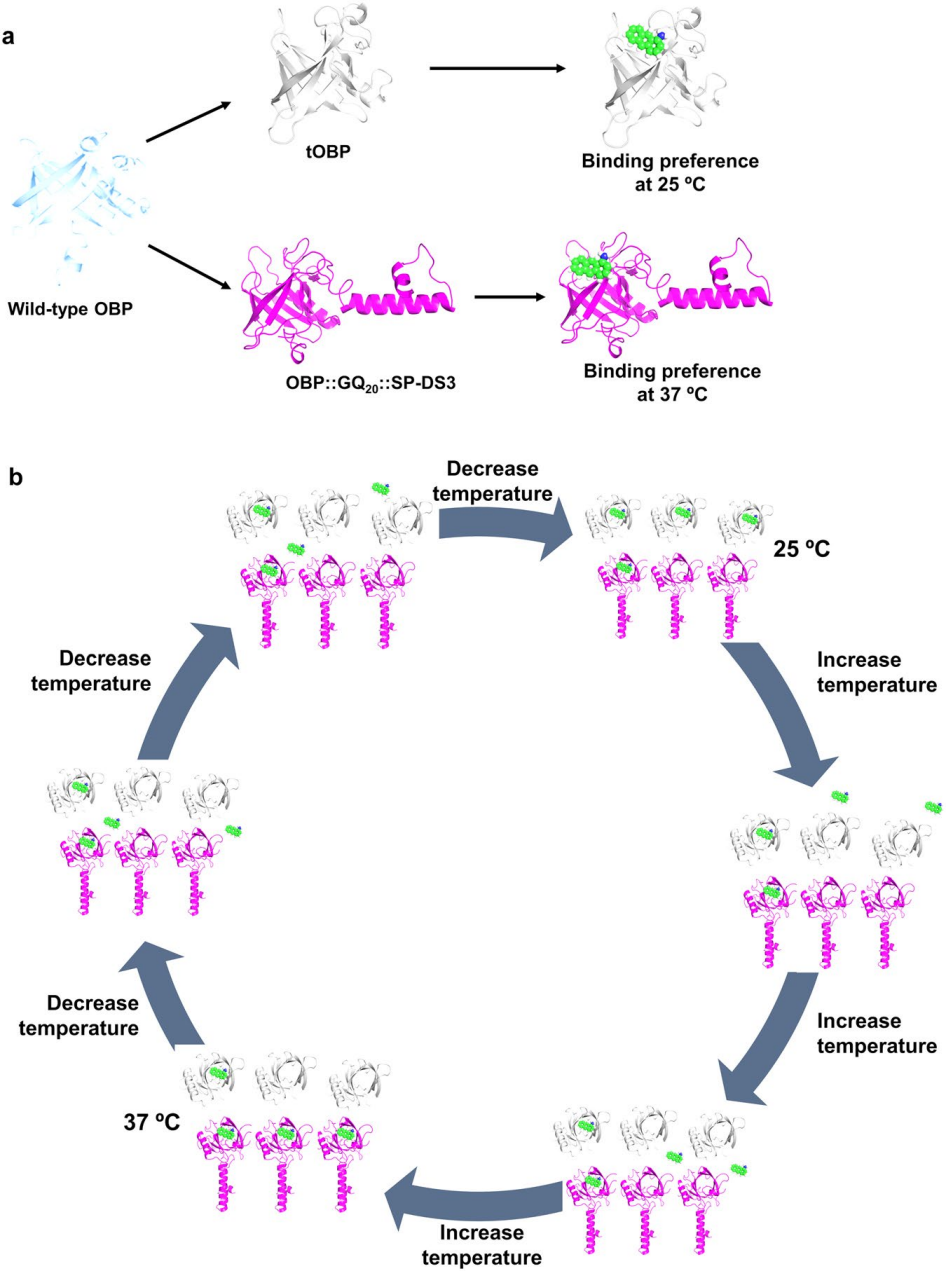
Opposite temperature-dependent affinities of tOBP and OBP::GQ_20_::SP-DS3 to 1-aminoanthracene (1-AMA). In (**a**) are presented the different binding affinities of tOBP and OBP::GQ_20_::SP-DS3. (**b)** Is the schematic presentation of OBP’s competitive temperature-dependent mechanism; tOBP is presented in grey; OBP::GQ_20_::SP-DS3 is presented in magenta and 1-AMA is presented in green.

Previous work revealed that the dissociation constants of 1-AMA to the wild-type OBP at 25 °C (Kd = 0.47 μM) and 37 °C (Kd = 0.37 μM) were similar^23^. The use of this protein on this study would not allow us to attain the proposed opposite temperature-dependent affinity towards 1-aminoanthracene. In the present work, we engineered two OBPs with opposite temperature-dependent affinities to evaluate the transfer of molecules in response to a thermal stimulus (Fig. 1). Truncated OBP (tOBP) resulted from the replacement of two phenylalanine residues at the binding pocket of OBP-I (F44A and F66A) and from the deletion of the first 16 residues of the N-terminal. These modifications were designed by modelling techniques which predict an opening of the binding pocket and a change from calyx-like structure to a channel. OBP::GQ_20_::SP-DS3 is the result of the fusion of OBP-I with the anchor peptide SP-DS3. A spacer of 20 repetitions of glycine-glutamine residues (GQ_20_) was included between the protein and the anchor peptide for conformational stability and molecular mobility. SP-DS3 was fused with OBP due to its ability to insert deeply into lipid membranes of liposomes^24^. The function of SP-DS3 peptide was not explored in this study. Sequences alignment of these new engineered proteins and the wild-type OBP-I are presented in supplementary data (Supplementary Fig. 1).

The affinity of the two engineered OBPs towards 1-aminoanthracene (1-AMA) was evaluated and compared at two distinct temperatures (25 and 37 °C). tOBP displayed higher affinity at 25 °C while 1-AMA bind preferentially to OBP::GQ_20_::SP-DS3 at 37 °C (Fig. 1a). Taking advantage of this distinct behavior we explored the affinity of OBPs’ to 1-AMA in a new temperature-dependent mechanism using temperature as the trigger. In a system equilibrated at 25 °C containing both OBPs separated by a permeable membrane, 1-AMA preferentially binds to tOBP. When the system temperature increases to 37 °C, 1-AMA (free and bind to tOBP) moves preferentially to OBP::GQ_20_::SP-DS3 (Fig. 1b).

To provide a fundamental understanding of the molecular mechanisms beyond the temperature-dependent affinities of tOBP and OBP::GQ_20_::SP-DS3 we performed circular dichroism (CD) studies and *in silico* experiments. We validated our model by performing experimental binding studies using 1-AMA as a model molecule to measure its binding association to OBPs at 25 and 37 °C.

For the processes and properties studied here, molecular modelling techniques are the perfect match to the experimental data collected. We demonstrated significant structural differences from the simulation of OBPs at different temperatures. In addition, we estimated the molecular docking sites and interactions of 1-AMA in the OBP designed proteins, and simulated the resulting complexes, demonstrating different preferences, depending on the tertiary structure and temperature.

## Results

### OBPs characterization

The engineered OBPs were characterized regarding purity and molecular weight by Matrix-Assisted Laser Desorption/Ionization with time-of-flight (MALDI-TOF). The data obtained by MALDI-TOF confirmed the monodisperse character of the proteins with the experimental molecular weight (17,832.53 Da for tOBP and 25,065.29 Da for OBP::GQ_20_::SP-DS3) in accordance with the theoretical values (Supplementary Fig. 2). The SDS-PAGE gels (Supplementary Fig. 2) also confirmed the purity of OBPs.

### Structural analysis

**Figure 2:**
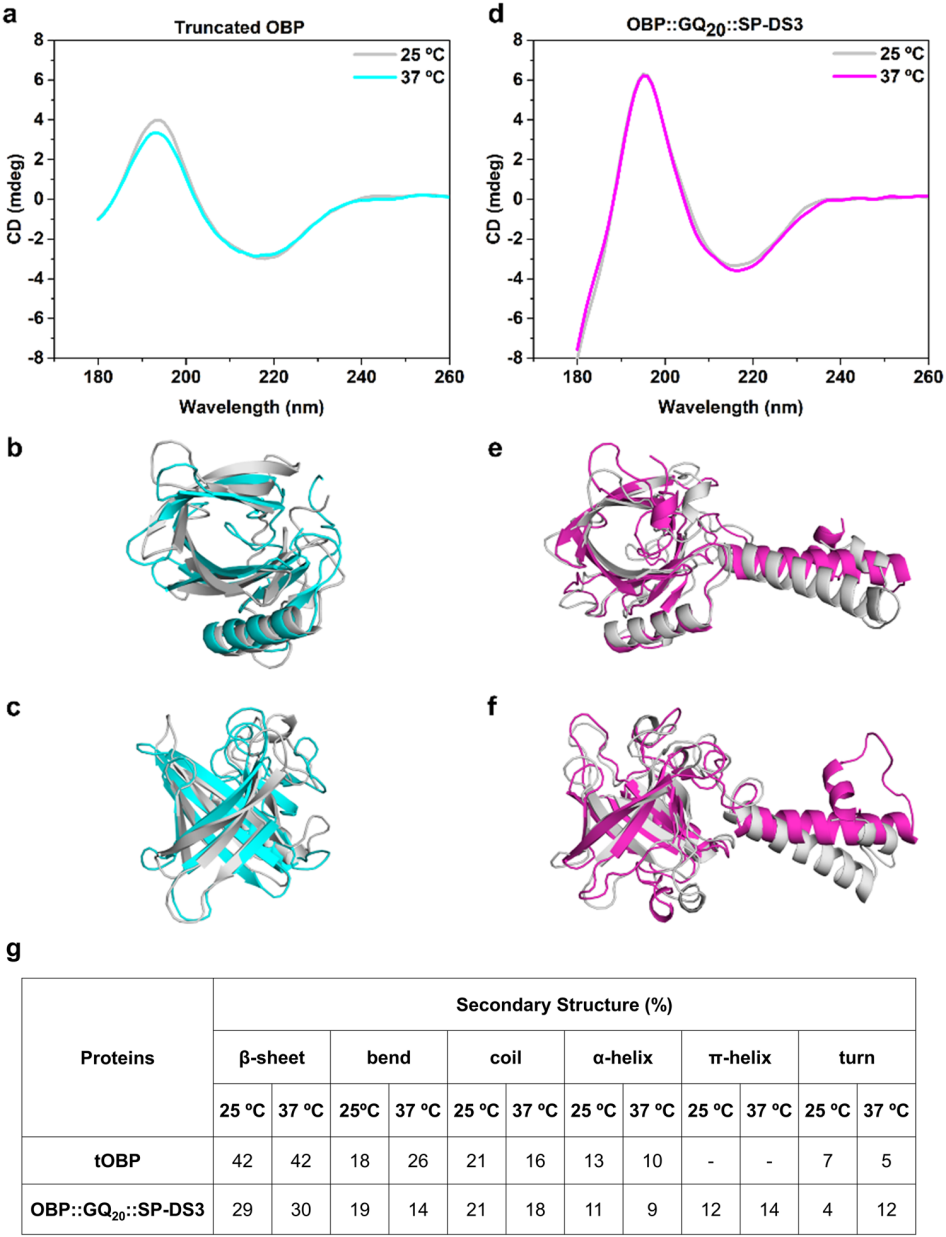
Structure of engineered OBP proteins analyzed by circular dichroism spectroscopy and molecular dynamics simulations. Secondary structure spectra determined by circular dichroism (CD) spectroscopy of tOBP (**a**) and OBP::GQ_20_::SP-DS3 (**d**); Top (**b**) and side (**c**) views of tOBP superimposed central structure; Top (**e**) and side (**f**) views of OBP::GQ_20_::SP-DS3 superimposed central structure. For all images, grey represents the structures at 25 °C, cyan and magenta represent the structures at 37 °C for tOBP and OBP::GQ_20_::SP-DS3, respectively. Percentage of secondary structures was calculated from DSSP^49^ (Dictionary of Secondary Structure in Proteins) method implemented on GROMACS^42^, for the central structures (CS) (**g**).

Dynamic changes in OBPs secondary structure triggered by temperature alterations was verified by circular dichroism (CD) spectroscopy and by molecular dynamic simulations (MD) at 25 °C and 37 °C. The CD spectra of the tOBP and the OBP::GQ_20_::SP-DS3 revealed the maximum and the minimum peaks around of 195 nm and 215 nm, respectively (Fig. 2a,d). This spectrum shape is characteristic of a fold with a high content of β-sheets which is in accordance with mammalian OBPs structure known to share a conserved folding pattern: an eight stranded β-barrel flanked by an α-helix at the C-terminal end of the polypeptide chain^12,13^. The CD spectra of both OBP variants confirmed the effect of the mutations on the protein structure comparing with the wild-type spectra (Supplementary Fig. 3). We observed an alteration of the tOBP and OBP::GQ_20_::SP-DS3 conformations at 25 and 37 °C, which was more evident for the tOBP. The less pronounced peaks displayed by this protein might be attributed to a more extended state of the β-sheets resulting from the partial unfolding of tOBP promoted by the deletion of the first 16 residues on the N-terminal. The fusion of the GQ_20_::SP-DS3 sequence with the wild-type OBP resulted in an increase of the helix content given by the presence of the coil/unordered SP-DS3 peptide^25^ and of the GQ_20_ linker structures^26,27^, as evidenced in the spectra (Supplementary Fig. 3).

The engineered OBPs present different spectra related with some structural differences which could affect the binding pocket conformation and affinity towards molecules. Comparing both CD spectra of tOBP at 25 and 37 °C, is evident a difference around 195 nm (Fig. 2a). At 25 °C, tOBP adopts a barrel structure in which the hydrogen bonds network becomes looser, promoting the opening of tOBP pocket cavity^12^. The CD spectra of OBP::GQ_20_::SP-DS3 at 25 and 37 °C show differences in the region of 215 nm (Fig. 2d). This variation can be associated to the increase of helix content from the SP-DS3 and GQ_20_ spacer^27^. From MD simulations, we took the central structure (CS), for each designed OBP, at each temperature, to compare the structural features among them. CS represents the most probable conformation for each protein under the simulation conditions. The convergence of simulated systems was traced by Root Mean Square Deviation (RMSD) analysis and compared with the previously simulated for wild-type OBP^28^. Supplementary Fig. 4 presents the RMSD plots of wild-type OBP, tOBP and OBP::GQ_20_::SP-DS3. RMSD of the engineered OBPs are in agreement with the experimental results, tOBP is more stable at 25 °C and OBP::GQ_20_::SP-DS3 at 37 °C.

Figure 2b,c corroborates the CD spectra results, i.e., when superimposed, the tOBP structures, demonstrated a more loose structure at 25 °C (grey structure) and a smaller barrel at 37 °C (cyan structure). OBP::GQ_20_::SP-DS3 displayed an opposite behavior (Fig. 2e,f), presenting a larger barrel at 37 °C (magenta structure), whereas at 25 °C a smaller barrel is observed (grey structure).

The quantitative analysis of the OBPs secondary structure (SS) content was determined by MD simulation. The Dictionary of Secondary Structure in Proteins (DSSP) method assigned secondary structure based on intra-backbone hydrogen bonds and main chain dihedrals. For each atom, the hydrogen bonds with best electrostatic energy are settled to a secondary structure. Figure 2g shows the percentages of secondary structure observed for the CS of both proteins. tOBP presents high content in β-sheets at both temperatures while the bend content is higher at 37 °C which might induced the closure of the barrel structure of the protein. The β-sheets content on OBP::GQ_20_::SP-DS3 is lower than on tOBP as this protein contains a higher helix content given by the linker region (20x GQ) and by the peptide SP-DS3 at the C-terminal. The GQ_20_ linker increases the percentage of helical content, with backbone atoms sample *i* → *i* + *4* and *i* → *i* + *5* hydrogen bond pattern simultaneously, i.e. counting α-helix and π-helix regions, respectively, or a switch of both SS. A π-helix is a more large secondary structure (the helix turn includes one more residue), and although very similar to α -helix, is most probable at higher temperatures, because the natural increase in entropy/kinetics, can lead to more loose structures. We also performed the same analysis based on the CD data and using the DichroWeb program^29^ (data not shown). The results are in good agreement with MD simulations, confirming the relation between the reduction of bends with the increase of binding-affinity. DSSP analysis assigned other SS content, based on the hydrogen bond patterns. Besides the typical β-sheet and α-helix SS, turns or π-helix, or even coil, may be attributed. Figure 2g indicates also the content of coil and turns. Turns can present a *i* → *i* + *3* or *i* → *i* + *4* hydrogen pattern, without enough amino acids to be a helix, being only a connection for helices or beta strands. It is expected a conserved content of β-sheets, due to the strength of the hydrogen bonds in this SS. Therefore changes in temperature do not impart the unfolding of the β-sheets while the content of coil and others SS may suffer variations.

### Binding affinity of engineered OBPs at 25 °C and 37 °C

**Figure 3:**
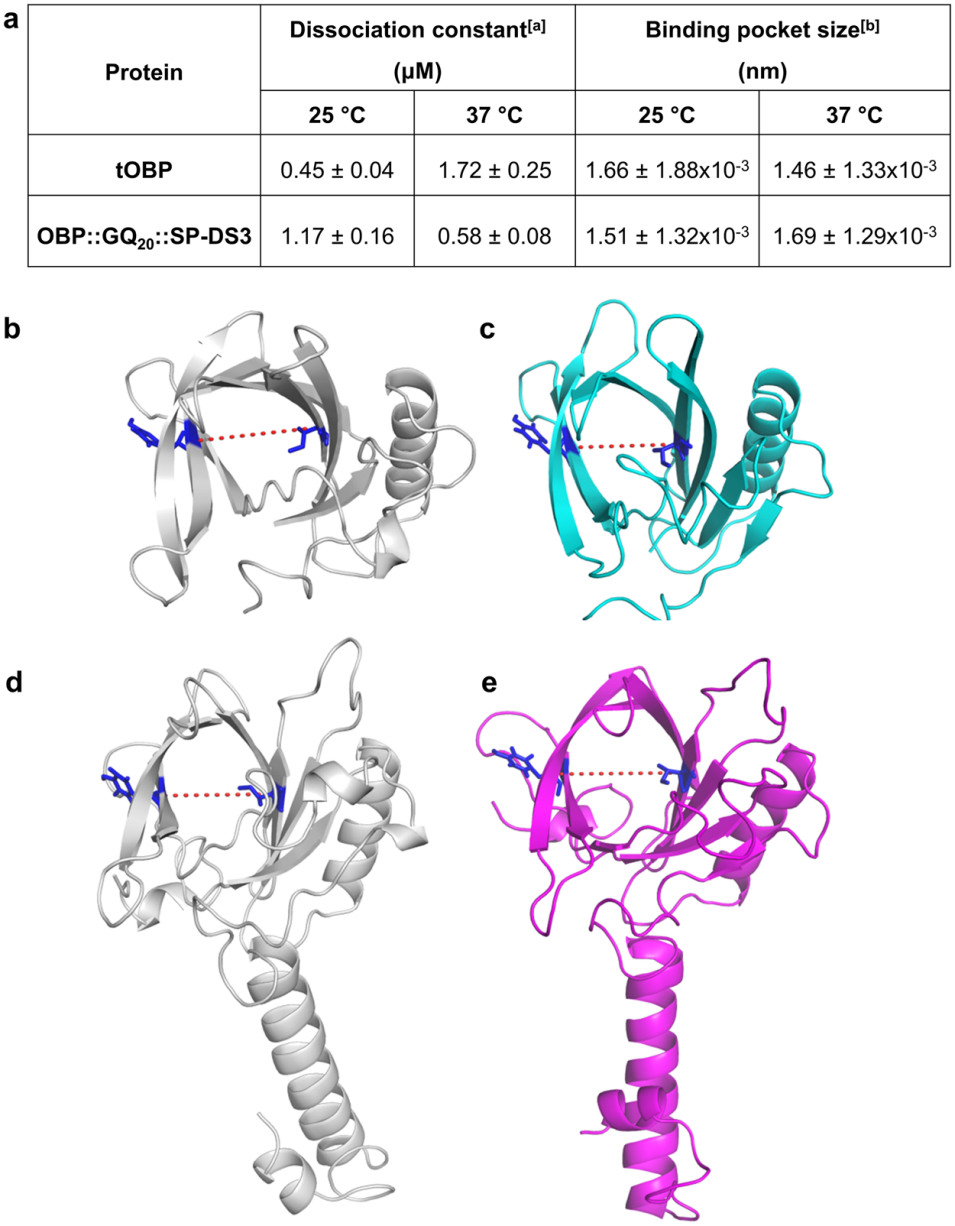
Dissociation constants and binding pocket size for tOBP and OBP::GQ_20_::SP-DS3. (**a**) Dissociation constants and binding pocket size of tOBP and OBP::GQ_20_::SP-DS3 at 25 and 37 °C. ^[a]^experimental data; ^[b]^molecular simulation data. Schematic presentation of tOBP (**b**,**c**) and OBP::GQ_20_::SP-DS3 (**d**,**e**) binding pocket measured at 25 and 37 °C. Protein binding pocket size was calculated between the center of mass of opposite residues in the β-barrel: Tyr52 and Ile100 highlighted in blue sticks, considering the central structures obtained from the last 15 ns MD simulations. Values are the mean ± SD of 2 independent experiments.

The binding properties of tOBP and OBP::GQ_20_::SP-DS3 were evaluated measuring their affinity towards 1-aminoantrance (1-AMA). The fluorescence-binding assays revealed opposite binding affinities for tOBP and OBP::GQ_20_::SP-DS3 depending on the temperature. At 25 °C, tOBP has a lower dissociation constant value (kd = 0.45 μM) than at 37 °C (1.72 μM), indicating a stronger binding of 1-AMA at 25 °C (Fig. 3a). An opposite performance is observed for OBP::GQ_20_::SP-DS3, demonstrating higher affinity at 37 °C (kd = 0.58 μM) than at 25 °C (kd = 1.17 μM) (Fig. 3a). The fluorescence binding curves are represented in the supplementary information (Supplementary Fig. 5). These differences might be related to the protein structure and the binding pocket rearrangement of the OBPs^30,31^. The secondary structure of tOBP at 25 °C, as verified by CD spectroscopy and MD simulations, present an extended barrel and a large binding pocket (1.66 ± 1.88 × 10^−3^ nm), acquiring some binding plasticity (kd = 0.45 μM). On the other hand, a more narrowed β-barrel and binding pocket is observed for tOBP at 37 °C (1.46 ± 1.33 × 10^−3^ nm), hindering a proper 1-AMA binding (kd = 1.72 μM) (Fig. 3b,c). The binding pocket of the OBP::GQ_20_::SP-DS3 at 25 °C is more closed (1.51 ± 1.32 × 10^−3^ nm) than at 37 °C (1.69 ± 1.29 × 10^−3^ nm), altering 1-AMA binding affinity (kd_25 °C_ = 1.17 μM; kd_37 °C_ = 0.58 μM) (Fig. 3d,e). The pocket size estimation considered opposite amino acids at the center of barrel core are demonstrated in Fig. 3b–e.

### Temperature competitive-binding of 1-AMA

**Figure 4:**
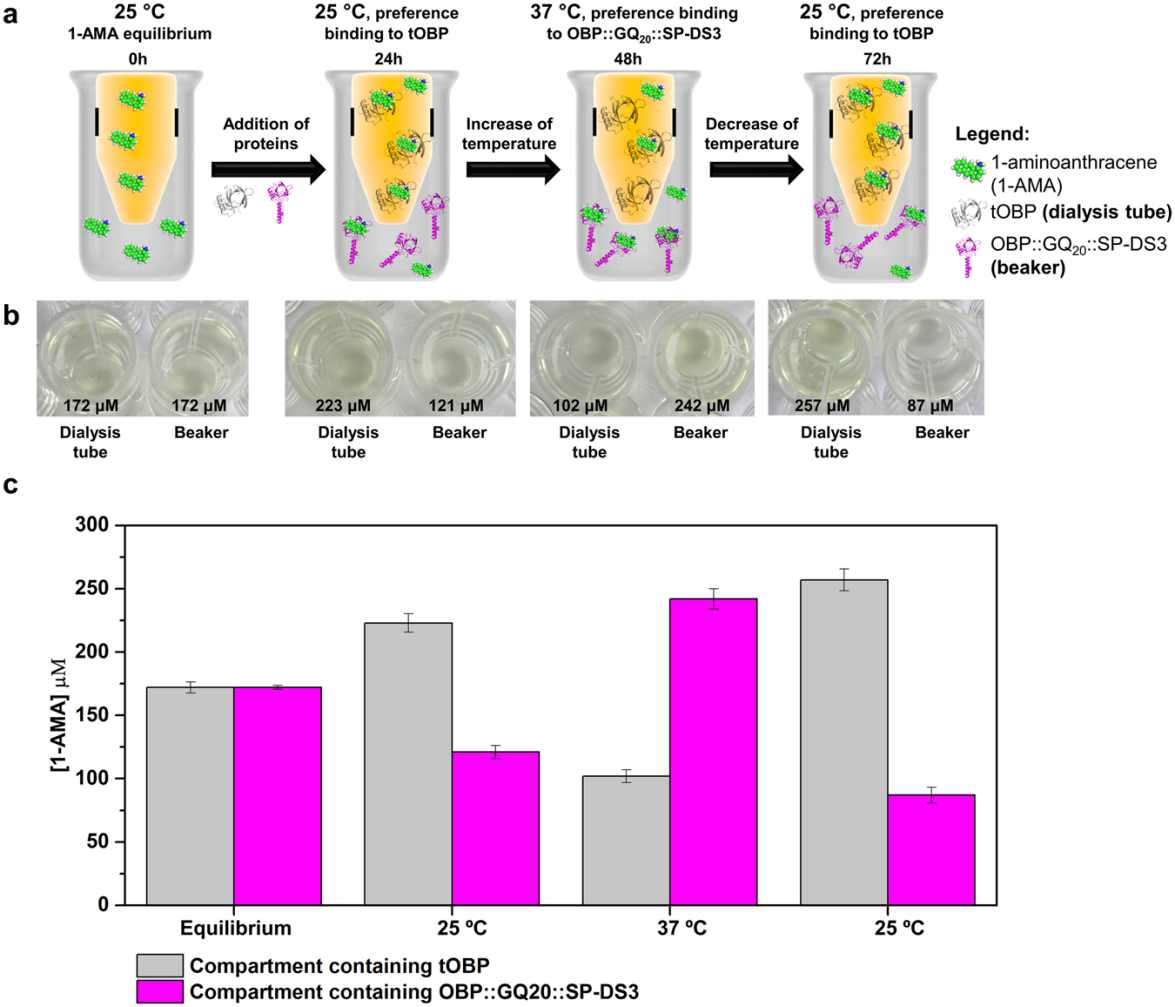
Fluorescence-binding assay of 1-AMA to tOBP and OBP::GQ_20_::SP-DS3. (**a**) Experimental layout for competitive binding evaluation; (**b**) visualization of 1-AMA in each compartment after variation of temperature; (**c**) amount of 1-AMA in each compartment after variation of temperature. Values are the mean ± SD of 2 independent experiments.

Considering the opposite affinities of tOBP and OBP::GQ_20_::SP-DS3 at 25 and 37 °C we developed a system to study the preferential movement of 1-AMA between both OBPs depending on the temperature. For that, we designed an experimental system composed by two compartments, a dialysis tube (cut-off 3.5 kDa) (allowing only the movement of 1-AMA) inside in a beaker. After equilibrium of 1-AMA (172 μM), the tOBP was added to the dialysis tube and the OBP::GQ_20_::SP-DS3 to the beaker. At 25 °C, the highest amount of 1-AMA was measured in the tOBP compartment (116 μM bind to the protein and 107 μM free). After increasing the temperature to 37 °C, 1-AMA preferentially moved to the beaker containing the OBP::GQ_20_::SP-DS3 (135 μM bind to the protein and 107 μM free). The temperature was further decreased to 25 °C promoting the preferential movement of 1-AMA again for the compartment containing the tOBP (237 μM bind to the protein and 20 μM free) (Fig. 4). These results are in accordance with fluorescence-binding assays previously reported **(**Fig. 3a).

### Molecular Docking and MD Simulations on OBPs/1-AMA complexes

**Figure 5:**
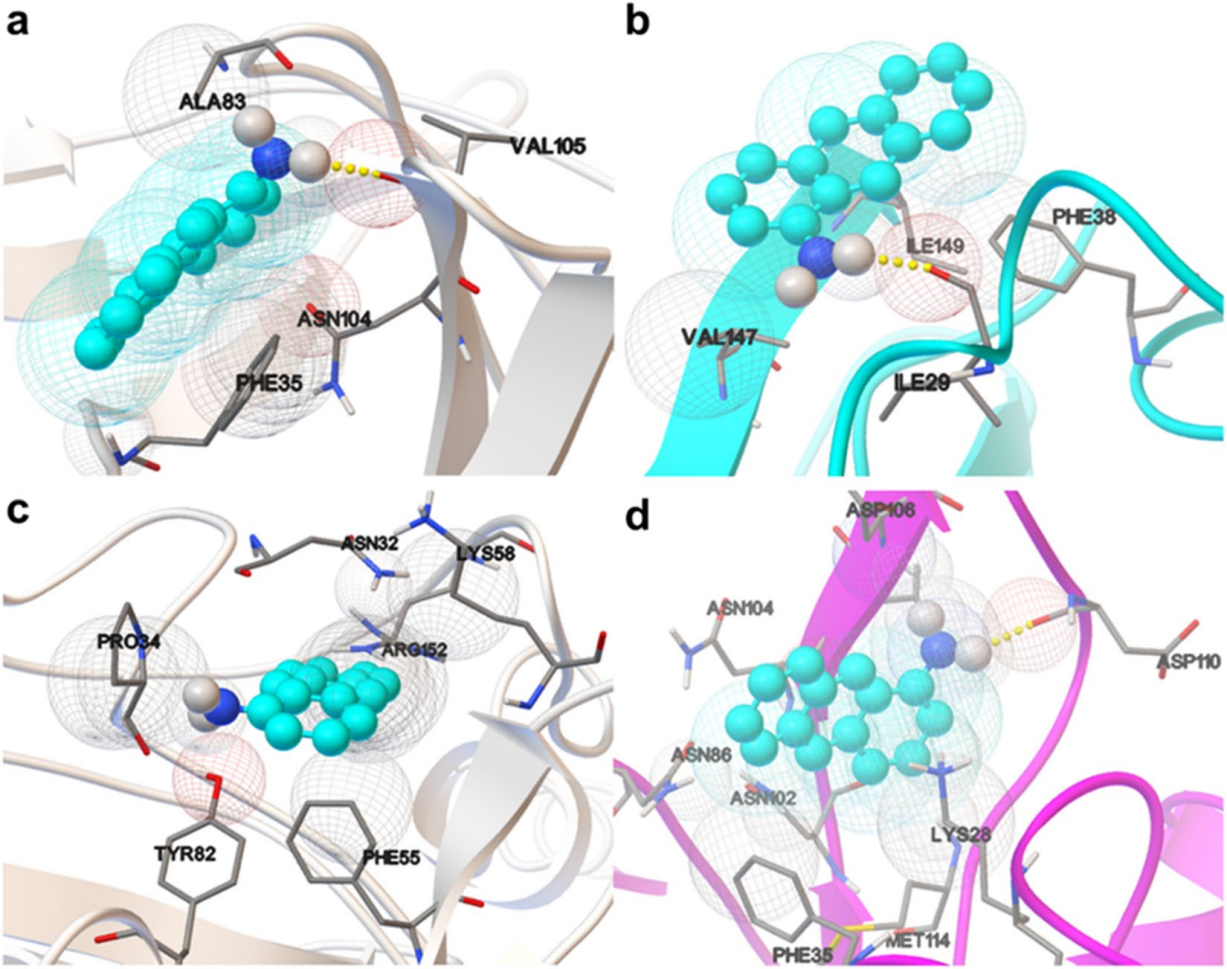
Interaction bind mode of 1-AMA to OBPs estimated through molecular docking with AutoDock Vina. 1-AMA interaction with tOBP at 25 °C (**a**) and at 37 °C (**b**). Ligand bind mode of 1-AMA to OBP::GQ_20_::SP-DS3 at 25 °C (**c**) and at 37 °C (**d**). 1-AMA ligand is presented in blue spheres and the amino acids involved in hydrogen bonds or van der Waals contacts, in CPK sticks representation.

Molecular docking provides detailed insights into the nature of ligand-protein interactions and the position of a ligand in the protein following the laws of statistical thermodynamics. The temperature-dependent affinity of 1-AMA to tOBP and OBP::GQ_20_::SP-DS3 (Fig. 4) was estimated by Gibbs binding energy (∆G) using AutoDock Vina at 25 °C and 37 °C. The energy values measured for both proteins at the two temperatures were similar, differing only in ~0.5–1.0 kcal/mol (data not shown). Consequently the docking results only give us a first insight about the binding mode preferences (position and interactions). The interactions between 1-AMA and the proteins, observed for the best docked pose (more negative ∆G value), are shown in Fig. 5. These docking positions were then submitted to 10 ns of MD simulation to follow the stability of each complex.

**Figure 6:**
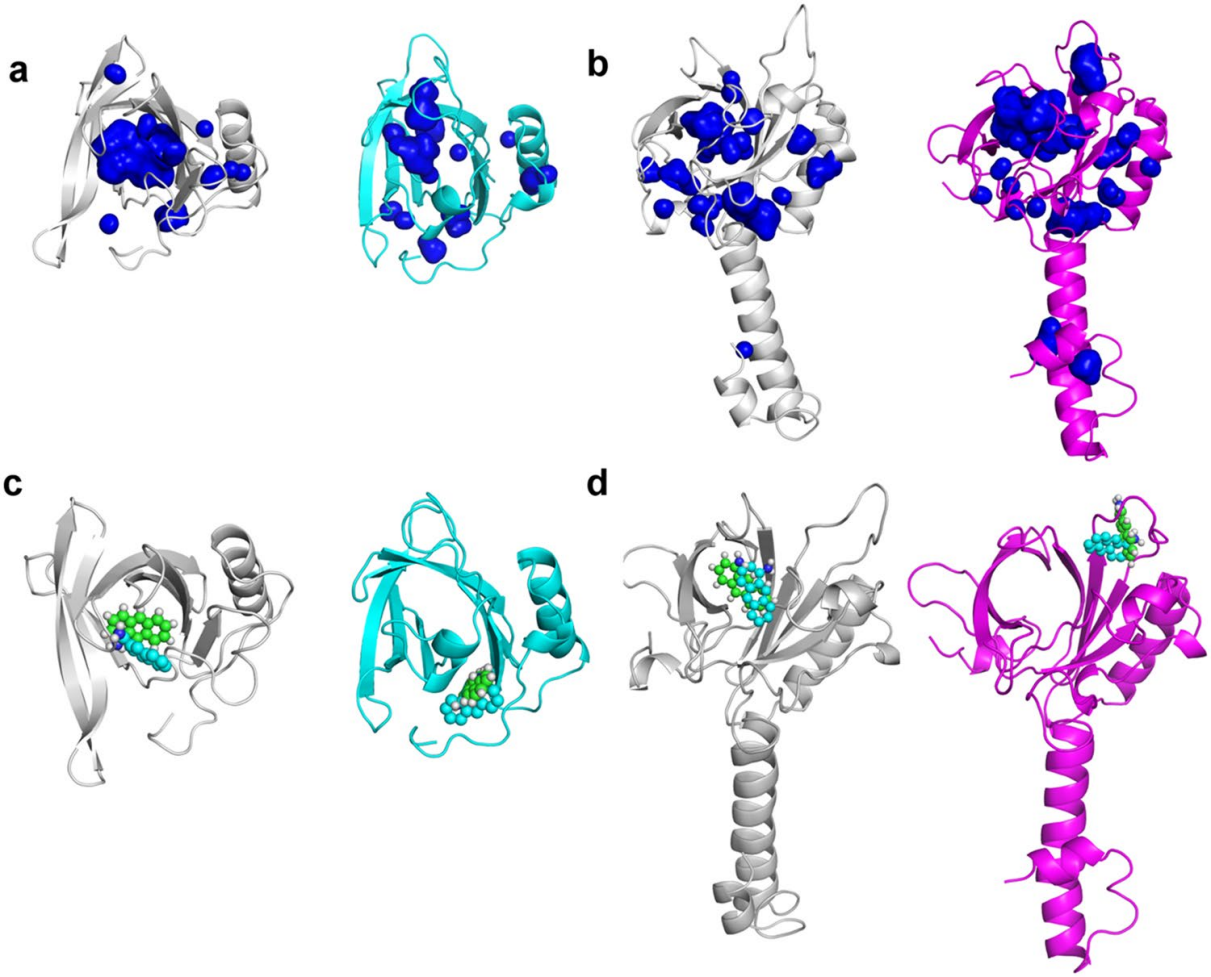
Comparison between docked position and middle structure from MD simulations. The position of 1-AMA was estimated through molecular docking (1-AMA in cyan spheres) and through MD simulation (1-AMA in green spheres). (**a**,**b)** Display the cavities estimated with PyMOL, in blue surface, for tOBP at 25 °C and 37 °C (**a**) and OBP::GQ_20_::SP-DS3, at 25 °C and 37 °C (**b**). In (**c**) is presented the most probable position for 1-AMA interaction with tOBP, at 25 °C and 37 °C, respectively. In (**d**) is shown the most probable bind mode of 1-AMA to OBP::GQ_20_::SP-DS3, at 25 °C and 37 °C, respectively.

The molecular modelling simulations revealed that OBPs undergo slight structural changes that may lead to the binding of 1-AMA to other sites rather than the preferential position. Looking at 1-AMA positions, we see that the ligand does not bind to the same region in all cases, for the best docked poses (similar poses were seen in all systems but ranked with less favored ∆G). PyMOL can display the cavities and pockets within the interior of a given molecule and looks at the complemental geometry and interactions between the ligand model and the protein. Using this tool we highlight the cavities that potentially can accommodate the 1-AMA ligand (Fig. 6). The docking results are in accordance with the PyMOL predictions and the experimental binding assays. 1-AMA binds preferentially to tOBP at 25 °C (Fig. 6a left) while to OBP::GQ_20_::SP-DS3, 1-AMA binds preferentially at 37 °C (Fig. 6b right), supported by the highest number of PyMOL docking possibilities.

Molecular docking has also been used as useful tool for analyzing the ligand interactions with the protein structure^32^. Thereby, using the AutoDock tools, we identified the amino acid residues of both OBPs which interact with 1-AMA, at 25 °C and 37 °C (Table 1). The main interactions occur with non-polar (hydrophobic) and polar amino acids. Some of these amino acids were previously described as residues of wild-type OBP-I interacting with odorants. Vincent *et al*. (2000) elaborated a list of residues of the OBP-I cavity which interact with different odors. Asn102 residue was described as interacting with three aromatic ligands: benzophenone (BZP), benzyl-benzoate (BZB) and 2-iso-butyl-3-metoxypyrazine (IBMP)^33^. Also, Met114 was recognized as being involved in the interaction between OBP-I and BZB^33^. Both of these residues were also identified in our study for OBP::GQ_20_::SP-DS3 at 37 °C. In another study, molecular simulation analysis identified the Tyr82 residue and nearby residues as forming the cavity entry. Tyr82 is in fact a residue conserved in many OBPs^32^. Additionally, the energetic analysis showed a high van der Waals interaction between the OBP and the odorants, representing more than 80% of the interaction energy compared with no strong hydrogen bond^32^. In the molecular dynamic study of Golebiowski *et al*. (2006), the Asn86 and Asn102 residues of OBP-I were identified as being involved in the binding of the 2,6-dimethyl-7-octen-2-ol (DHM)^32^. Nagnan-Le Meillour *et al*.^34^ performed molecular studies from crystallized pig OBP complexed with undecanal (UND). The results showed that Tyr82 and Phe35 residues participate in the binding process. Phe55 was also identified but in a less extent^34^. All of these residues are located at the border of the binding pocket and were identified in our study. Meillour *et al*.^34^ confirmed the simulation results by performing mutations in Tyr82 and Phe35. Fluorescence spectroscopy results indicated that both residues are involved in the binding of 1-AMA since the singles and double mutants were unable to bind 1-AMA^34^. The study revealed that the Phe38 residue is also involved in the binding to UND. Some charged residues (Lys28, Asp110) participate on the dissociation via their side chain hydrogen atoms^34^. These amino acids, Lys28 and Asp110, were also identified in our simulation analysis. Our study is a step further the existing knowledge since we describe other residues involved in the binding of 1-AMA to OBPs (Table 1, in bold).

**Table 1:** Amino acid residues of tOBP and OBP::GQ_20_::SP-DS3 involved in the 1-AMA binding.

	tOBP		OBP::GQ_20_::SP-DS3	
	25 °C	37 °C	25 °C	37 °C
Hydrogen Bond	VAL105	ILE29	None	ASP110
van der Waals contact	PHE35**/ALA83/ASN104**	PHE38**/VAL147/ILE149**	**ASN32/PRO34/**PHE55**/LYS58/**TYR82**/ARG152**	LYS28/PHE35/ASN86/ASN102/**ASN104/VAL105/ASP106/**MET114

The analysis was performed using the AutoDock Vina at 25 and 37 °C. In bold are described the new residues involved in the binding of 1-AMA to OBPs yet not identified in literature.

From 10 ns MD simulations on docking poses, we observed that the tOBP at 37 °C and OBP::GQ_20_::SP-DS3 at 25 and 37 °C, conserved the docked pose and the interactions with 1-AMA. Interestingly, for tOBP at 25 °C was verified a spontaneous movement of the 1-AMA to the interior of the β-barrel. This result is in great agreement with experimental data, which consider this protein as the most able to carry the ligand at this temperature (kd = 0.45 μM at 25 °C). Figure 6 shows a superposition of docked and simulated positions for 1-AMA, for the two proteins at both temperatures.

We see in Fig. 6c,d that MD simulation generates a middle structure around the docked position. In these cases, 1-AMA moves in the same binding region sampling the docked position along the simulation, although with a central structure, derived from the simulation in a slightly different position. The stability of the complex was then proved, using MD simulations, in these three cases: OBP::GQ_20_::SP-DS3/1-AMA, at 25 °C and 37 °C, and tOBP/1-AMA at 37 °C. The interactions with the same group of amino acids observed by docking (Table 1) are sampled along the simulation time. In the case of 1-AMA sampled in complex with tOBP at 25 °C, the ligand demonstrates a spontaneous movement toward the interior of the β-barrel. This fact might be an indication that under dynamics, the system is able to rearrange to better accommodate 1-AMA, protecting this hydrophobic ligand from the water environment.

To give an estimation about the free energy vs time interaction, we use the g_mmpbsa tool^35^ to follow the binding energy along the simulated complexes trajectories. Supplementary Fig. 6 shows the ligand-protein binding energy, for both systems at both temperatures, supporting the preference of 1-AMA for tOBP at 25 °C and for OBP::GQ_20_::SP-DS3 at 37 °C.

## Discussion

We have constructed two new OBPs based on the sequence of OBP-I aiming different goals. tOBP was engineered to impart selective porosity while OBP::GQ_20_::SP-DS3 was designed to anchor to lipid membranes. Our data on the ability to bind 1-AMA revealed that tOBP and OBP::GQ_20_::SP-DS3 had opposite binding affinities depending on the temperature. Based on this finding we have explored the thermo-responsive behavior of the engineered OBPs to study the movement of 1-AMA between them using temperature as trigger. We have observed that the binding affinity of the two OBPs was directly related with their structural conformation induced by temperature. The dissociation constants confirmed the high affinity of 1-AMA to tOBP at 25 °C (kd = 0.45 μM) and to OBP::GQ_20_::SP-DS3 at 37 °C (kd = 0.58 μM). The binding pocket size and the bend content were considered essential to understand the thermo-responsive behavior of the OBPs. Both parameters were evaluated by CD spectroscopy and MD simulations confirming their fundamental contribution to OBPs behavior. The average size of the pocket is directly related with OBPs’ binding affinity while the bend content is inversely proportional. At 25 °C tOBP presents a wider pocket size and a more relaxed structure than OBP::GQ_20_::SP-DS3, thus showing higher affinity to 1-AMA. At 37 °C, we observed a higher bend content indicating conformational alterations of tOBP resulting in a smaller pocket size, therefore decreasing the binding of 1-AMA. At this temperature, OBP::GQ_20_::SP-DS3 presented an opposite behavior, lower bend content and larger pocket size, resulting in an higher binding of 1-AMA compared with tOBP.

We found a temperature-dependent affinity competition between the two OBPs when placed in the same system. When triggered by temperature there was a reversible displacement and movement of 1-AMA from one OBP to the other. Docking experiments also characterized the 1-AMA binding preference and locations of the ligand in the two engineered proteins. The MD data are in agreement with the experimental results. The 10 ns of MD simulation confirmed the docking results for tOBP/1-AMA at 37 °C and OBP::GQ_20_::SP-DS3/1-AMA, at 25 °C and 37 °C. 1-AMA binding to tOBP at 25 °C shows a spontaneous phenomenon of insertion in the β-barrel. The modeling techniques have shown variations on the binding site, resulting from the structural and dynamic profile conferred by temperature changes. This suggests that OBP-based proteins are able to adjust and offer new binding locations at the entrance of the pocket, while keeping their carrier function.

In summary, the engineered OBPs explored in this work showed tunable affinities upon temperature changes. This feature was described for the first time for this class of proteins opening space to the exploitation of a new class of functional materials.

## Methods

### Reagents

Tris-base, imidazole, sodium phosphate and sodium chloride were available from Sigma-Aldrich. 1-aminoantrance (1-AMA) was purchased from TCI chemicals. Nickel Magnetic Beads for His Tag Protein Purification was available from Biotool, Bimake. Molecular weight Precision Plus Protein^TM^ standards were purchased from BioRad. Dialysis tubes Midi 3500, capacity 50–800 μL, MWCO 3.5 kDa (Pur-A-Lyzer™ Midi Dialysis Kit) were available from SIGMA. All other reagents were acquired from Sigma-Aldrich and used as received.

### Proteins production and purification

Two proteins (tOBP and OBP::GQ_20_::SP-DS3) based on the sequence of OBP-I (PDB code 1DZK) were engineered. Truncated OBP (tOBP) resulted from the replacement of two phenylalanine residues at the binding pocket of OBP-I (F44A and F66A) and from the deletion of the first 16 residues of the N-terminal. OBP::GQ_20_::SP-DS3 is the result of the fusion of OBP-I with the anchor peptide SP-DS3 (DRDDQAAWFSQY) and a linker of 20 repetitions of glycine-glutamine residues (GQ_20_). The OBP genes were synthetized by GenScript and cloned in pET-28a plasmid. OBPs were produced in *Escherichia coli* BL21(DE3) in Lysogeny broth (LB) with induction-cell at an optical density of 0.5–0.6 with Isopropyl β-D-1-thiogalactopyranoside (IPTG). Cells were harvest by centrifugation at 7,000 × g, for 5 min at 4 °C, resuspended in phosphate buffer (20 mM sodium phosphate, 500 mM NaCl, pH 7.4) supplemented with 10 mM of imidazole and lysed by sonication (40%, 3.0 s ON, 9.0 s OFF for 10 min) in sonicator vibracell^TM^ SONICS. Soluble and insoluble fractions were separated by centrifugation at 12,000 xg, for 30 min at 4 °C. The soluble fraction was purified through Nickel magnetic beads with specificity to His-tag present in the protein’s N-terminal. To remove the presence of high salts content and imidazole after purification the samples were dialyzed for 3 days, at 4 °C against ultrapure water.

### MALDI-TOF mass spectrometry

Mass of OBP proteins was verified by Matrix-Assisted Laser Desorption/Ionization with time-of-flight (MALDI-TOF) using sinapic acid (SA) as matrix (≥99.5%). The mass spectra were acquired on an Ultra-flex MALDI-TOF mass spectrophotometer (Bruker Daltonics GmbH) equipped with a 337 nm nitrogen laser. A double layer deposition was used to analyze the OBPs. For this, a saturated solution of SA in ethanol, was deposited in the ground steel plate until dry. Each sample, previously dissolved in TA30 (30% acetonitrile/70% TFA), was mixed (1:1) with a saturated solution of SA in TA30. A volume of 2 μL of each mixture was spotted onto the ground steel target plate (Bruker part n° 209519) and analyzed using the reflective positive mode.

### Circular dichroism (CD) spectroscopy

The structural state of OBPs was investigated by circular dichroism (CD) spectroscopy, using a Jasco J-1500 spectropolarimeter equipped with a temperature controller. Far-UV CD spectra were recorded in a 1-mm-path-length cell from 260 to 180 nm with a 1 nm resolution and at a scan speed of 20 nm/min. CD spectra were recorded at 25 °C and 37 ± 0.1 °C, using 10 μM as a fixed concentration. Baseline was recorded with the same buffer of the samples (5 mM phosphate buffer, pH 7.5) and subtracted to the protein spectra. Final spectra were generated by the average of three scans for each sample.

### Fluorescence binding studies at 25 °C and 37 °C

The ligand binding experiments were performed by direct titration with 1-AMA, as reported by Silva *et al*. (2013)^23^, at 25 °C and 37 °C. Briefly, the fluorescent probe 1-AMA was dissolved in 95% ethanol as 1 mM stock solutions. Successive increasing ligand concentrations (in buffer solution) were added to 1 μM of proteins and incubated at 25 and 37 ± 0.1 °C, for 15 minutes, in a microplate spectrofluorometer (BioTek Synergy MX) equipped with a temperature controller, with slits set at 5 nm bandwidth. The fluorescence emission spectra were recorded in three independent experiences and read in triplicate, measuring the OBP-ligand complex formation by the increase of emission intensity at 481 nm when excited at 295 nm^23,36^. Dissociation constants (kd) were calculated from a plot of fluorescence intensity versus concentration of ligand, obtained with a standard non-linear regression method, described by Malpeli *et al*.^37^.

### Temperature competitive-binding of OBPs to 1-AMA

To study the competition between tOBP and OBP::GQ_20_::SP-DS3 for 1-AMA we developed a system constituted by the two OBPs separated by a dialysis membrane with a cut-off only permeable to 1-AMA (3.5 kDa). tOBP was placed inside the dialysis tube and OBP::GQ_20_::SP-DS3 was placed outside, in a beaker. Initially, 1 mM of 1-AMA was added to the dialysis tube and incubated at 25 °C until equimolar equilibrium (172 μM). Further, 172 μM of the tOBP was added inside of the dialysis tube and 172 μM of OBP::GQ_20_::SP-DS3 was added in the beaker. After incubation at 25 °C for 24 h the concentration of 1-AMA in both compartments was measured by fluorescence spectroscopy. Afterwards the temperature of the system was increased until 37 °C and maintained during 24 h. After this period 1-AMA concentration was measured in both compartments. The temperature of the system was lowered to 25 °C, and the concentration of 1-AMA was again measured after 24 h of incubation. The concentration of 1-AMA was determined by measuring the fluorescence of free 1-AMA fractions in the dialysis tube and in the beaker at 600 nm (λ_ex_ = 295 nm) and replacing the experimental value in the calibration curve of fluorescence versus 1-AMA concentration. All steps were visually evaluated by photographic record. Measurements were recorded in two independent experiments and the results were expressed as mean value ± standard deviation (SD).

### Statistical analysis

The values reported in the circular dichroism spectra were generated by the average of three scans for each sample. The CD data were fitted with a Boltzmann sigmoidal line shapes.

### Molecular Dynamics Simulations

tOBP and OBP::GQ_20_::SP-DS3 were designed with PyMOL^38^, based on the OBP experimental structure 1DZK^33^, from PDB (Protein Data Bank)^39^ and using an equilibrated wild-type OBP. For our previous work on wild-type OBP^28^, we simulated OBP-I for 60 ns. We took a representative structure from this simulations to proceed with the necessary changes to the construction of the OBP-based proteins proposed here. The wild-type form is very stable and conserved (see RMSD, in Supplementary information, Supplementary Fig. 4). Both proteins were modeled in water with the simple point charge (SPC) water model in an octahedral box with a hydration layer of at least 1.5 nm between the peptide and the walls. Na+ ions were added to neutralize the simulation boxes. One stage of energy minimization was performed using a maximum of 50,000 steps with steepest descent algorithm for both structures. The systems were initialized in a NVT ensemble, using V-rescale^40^ algorithm, with the coupling constant τ_T_ = 0.10 ps, to control temperature at 298 K (25 °C) and 310 K (37 °C), i.e. each system was settled to generate two independent runs at each temperature. After that, a NPT initialization step was performed, with V-rescale and Parrinello-Rahman barostat^41^ algorithms to couple temperature and pressure at 298 K/310 K and 1 atm respectively. We used the following coupling constants: τ_T_ = 0.10 ps and τ_P_ = 2.0 ps. Position restraints (with force constant of 1000 kJ·mol-1·nm-2) were applied to all protein heavy atoms in initialization. 20 ns of MD simulations were performed for each system, without position restraints, and with the same NPT ensemble described above (RMSD for the new OBPs remains within an acceptable range and similar to the observed for wild-type OBP; see Supplementary Fig. 4).

All simulations were performed using the GROMACS 4.5.4 version^42,43^, within the GROMOS 54a7 force field (FF)^44^. The Lennard-Jones interactions were truncated at 1.4 nm and using particle-mesh Ewald (PME)^45^ method for electrostatic interactions, also with a cut-off of 1.4 nm. The algorithm LINCS^46,47^ was used to constrain the chemical bonds of the peptides and the algorithm SETTLE^48^ was used in the case of water.

### MD simulations analysis

MD simulations were performed to equilibrate the two engineered proteins at 25 °C and 37 °C. From MD simulations in water, at 25 °C and 37 °C, we computed the central structure (CS) of each engineered protein, for the last 15 ns of simulation time. These conformations minimize the RMSD variance when fitted against all other conformations of the trajectory, corresponding to the most populated conformation of the simulation. For these systems, we fitted the backbone and calculated the backbone RMSD (Root Mean Square Deviation). Then from the RMSD matrix, we extracted the most representative conformation of each simulation. The Secondary Structure (SS) profile was also computed by the Dictionary of Secondary Structure in Proteins (DSSP) method, from Kabsch and Sander (1983)^49^ that allows to calculate the percentage of each SS founded along the simulation time, using the hydrogen bond pattern. Both tools are implemented on GROMACS.

The average binding pocket size was determined measuring the distance between the center of mass of tyrosine 52 (Tyr52) and isoleucine 100 (Ile100), along time. These amino acids are in opposite directions and symmetrically arranged in the β-barrel for all protein models.

### Molecular Docking and MD Simulations on 1-AMA/OBPs complexes

Docking experiments were performed using AutoDock Vina^50^ and prepared with the AutoDock Tools Software^51^. We used the Central Structures at each temperature, obtained from the first round of MD simulations for the docking experiments. AutoDock Vina requires a grid spacing of 1 Å, generating boxes with approximately 28 × 30 × 30 grid points, for all systems. It uses a combination of scoring function and an optimization algorithm, being fastest in predict poses. We used an exhaustiveness of 15, num_modes = 50 and energy range = 3. The amino acid residues of the proteins that interact with the 1-AMA were identified through the AutoDock tools at 25 °C and 37 °C. However, as the binding energies differ only in ~0.5–1.0 kcal/mol between the systems, at both temperatures, we look at docking results only to see the interaction binding mode and extract the best binding pose to proceed to MD simulations of the complexes.

10 ns of MD simulations were performed for the 4 complexes obtained in the previous step. The same protocol applied to the free proteins simulations was used to simulate the complexes. The stability of each complex was followed through directly visualization of each trajectory. We also generate a central structure from these simulations, to serve as the most representative complex conformation.

From the 10 ns MD simulations, we performed MM_PBSA calculations (Molecular Mechanics Poisson-Boltzmann Surface Area) to estimate the binding energy of the four AMA/OBPs complexes along time, using g_mmpbsa tool^35^. We set the temperature for these calculations according to the simulations temperature, i.e. 25 °C or 37 °C. These results are presented as running average curves in Supplementary Fig. 6.

## Electronic supplementary material

Supplementary information
