# Supplementary material for: Two Engineered OBPs with opposite temperature-dependent affinities towards 1-aminoanthracene
Source: Sci Rep. 2018 Oct 4;8:14844. doi: 10.1038/s41598-018-33085-8 (PMC6172251; doi:10.1038/s41598-018-33085-8)
Supplement: Supplementary file 1 — Supplementary information [file 41598_2018_33085_MOESM1_ESM.pdf]

Supplementary information

Two Engineered OBPs with opposite temperature-dependent affinities towards 1-aminoanthracene

Filipa Gonçalves[1], Tarsila G. Castro[1], Nuno G. Azoia[1], Artur Ribeiro[1], Carla Silva[1], Artur Cavaco-Paulo[1*]

```
OBPwt              QEPQPEQDPFELSGKWITSYIGSSDLEKIGENAPFQVFMRSIEFDDKESKVYLNFFSKEN
OBP::GQ20::SP-DS3  QEPQPEQDPFELSGKWITSYIGSSDLEKIGENAPFQVFMRSIEFDDKESKVYLNFFSKEN
tOBP               ----------------ITSYIGSSDLEKIGENAPFQVFMRSIEADDKESKVYLNFFSKEN
                                   *************************** ****************

OBPwt              GICEEFSLIGTKQEGNTYDVNYAGNNKFVVSYASETALIISNINVDEEGDKTIMTGLLGK
OBP::GQ20::SP-DS3  GICEEFSLIGTKQEGNTYDVNYAGNNKFVVSYASETALIISNINVDEEGDKTIMTGLLGK
tOBP               GICEEASLIGTKQEGNTYDVNYAGNNKFVVSYASETALIISNINVDEEGDKTIMTGLLGK
                   ***** ******************************************************

OBPwt              GTDIEDQDLEKFKEVTRENGIPEENIVNIIERDDCPA-----------------------
OBP::GQ20::SP-DS3  GTDIEDQDLEKFKEVTRENGIPEENIVNIIERDDCPAGQGQGQGQGQGQGQGQGQGQGQG
tOBP               GTDIEDQDLEKFKEVTRENGIPEENIVNIIERDDCPA-----------------------
                   *************************************

OBPwt              -----------------------------
OBP::GQ20::SP-DS3  QGQGQGQGQGQGQGQGQDRDDQAAWFSQY
tOBP               -----------------------------
```

Supplementary Figure 1 | Sequences alignment of OBP wt, tOBP and OBP::GQ_{20}::SP-DS3**..** The alignment was performed using the CLUSTAL O (1.2.4) multiple-sequence alignment program. In red are highlighted the alanine residues mutated in tOBP; in blue is indicated the linker composed by glycine and glutamine repetition added to OBP::GQ_{20}::SP-DS3 protein; in green is highlighted the SP-DS3 peptide.

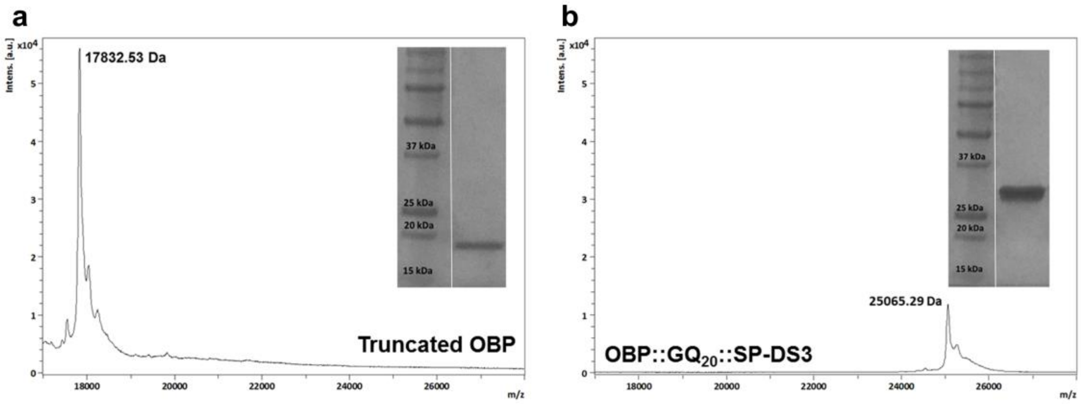

c

Protein	Theoretical mass	m/z
tOBP	17952,79	17832,53
OBP::GQ_{20}::SP-DS3	25189,19	25065,29

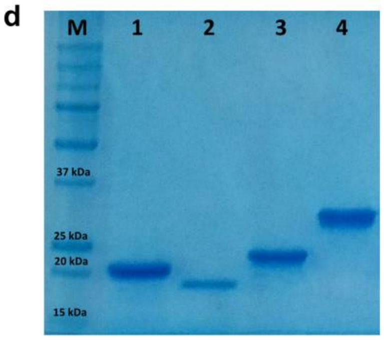

Supplementary Figure 2 | MALDI-TOF and SDS-PAGE gel of tOBP (a) and OBP::GQ_{20}::SP-DS3 (b). Theoretical mass and the m/z values are indicated in (c). In (d) is shown the original gel from that was grouping the gels indicated in (a) and (b), being the truncated OBP the run of the line 2 and the OBP::GQ_{20}::SP-DS3 the run of the line 4.

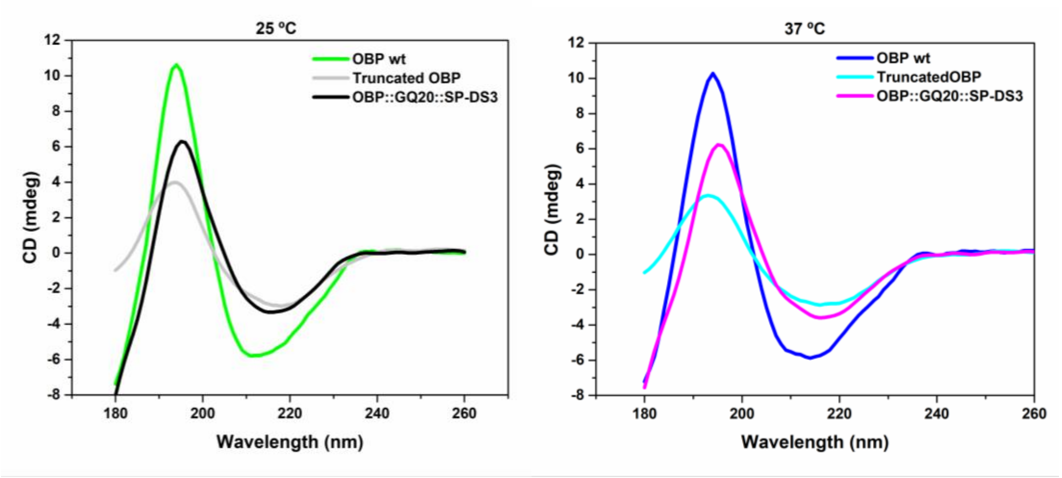

Supplementary Figure 3 | Circular dichroism (CD) spectra of wild-type OBP, truncated OBP and OBP::GQ_{20}::SP-DS3, at 25 ºC and 37 ºC. Final spectra were generated by the average of three scans for each sample.

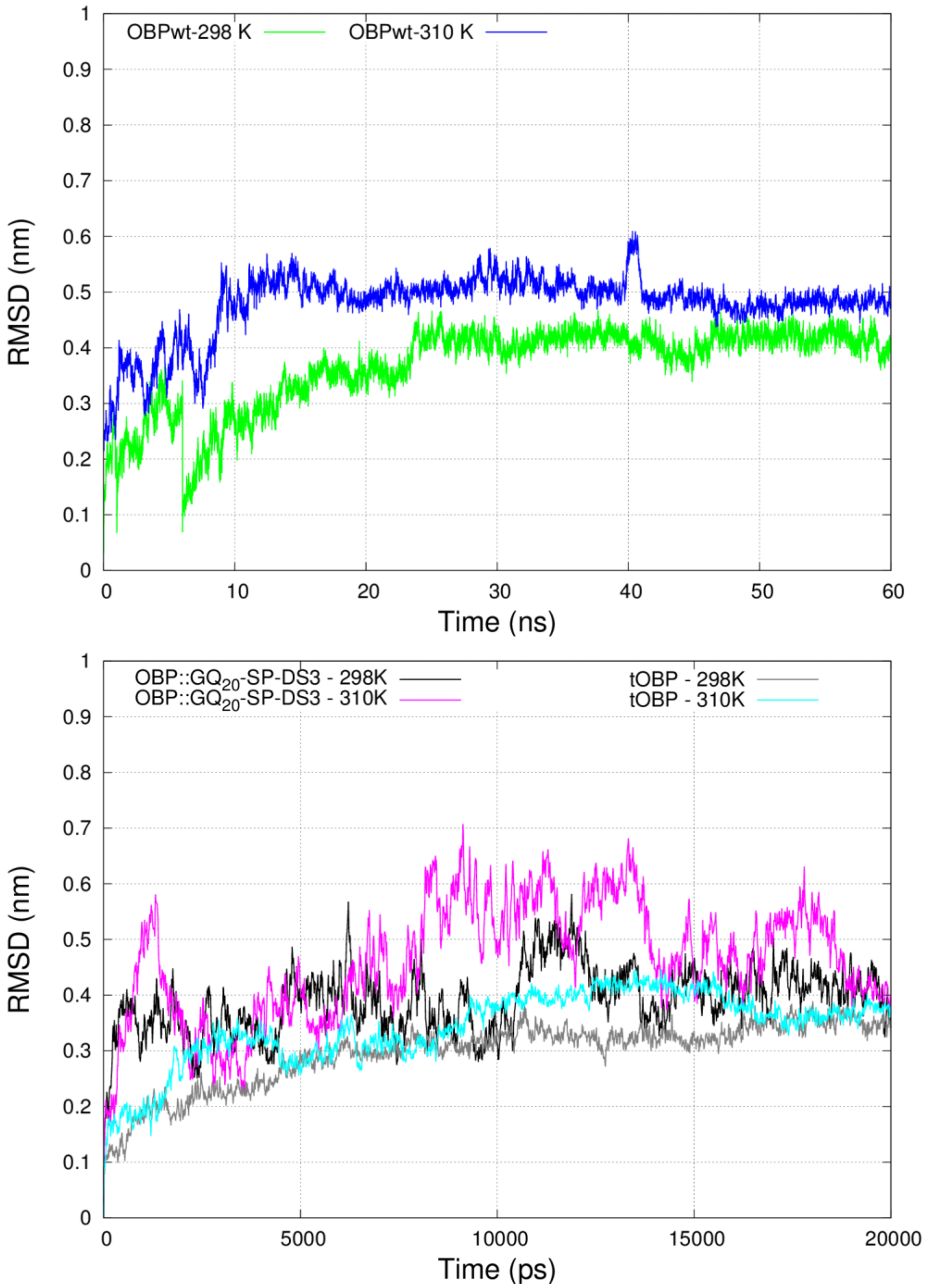

Supplementary Figure 4 | Backbone RMSD for wild-type OBP (top) along 60 ns MD simulation and for the engineered OBPs, along 20 ns of simulation time. Backbone atoms were fitted in all cases.

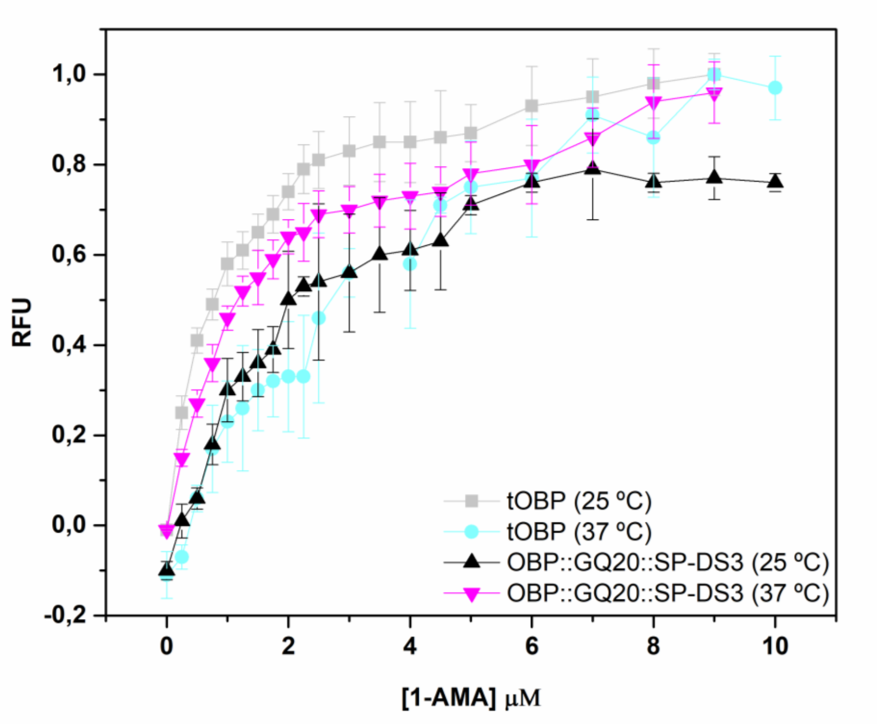

Supplementary Figure 5 | Binding curves of tOBP and OBP::GQ_{20}::SP-DS3 obtained by measuring the fluorescence of 1 μM protein in 50 mM Tris–HCl, pH 7.5, at equilibrium with several concentrations of 1-aminoanthracene (1-AMA). The dissociation constants were obtained at two temperatures, 25 °C and 37 °C, by mathematical fitting of data. Values are the mean ± SD of 3 independent experiments.

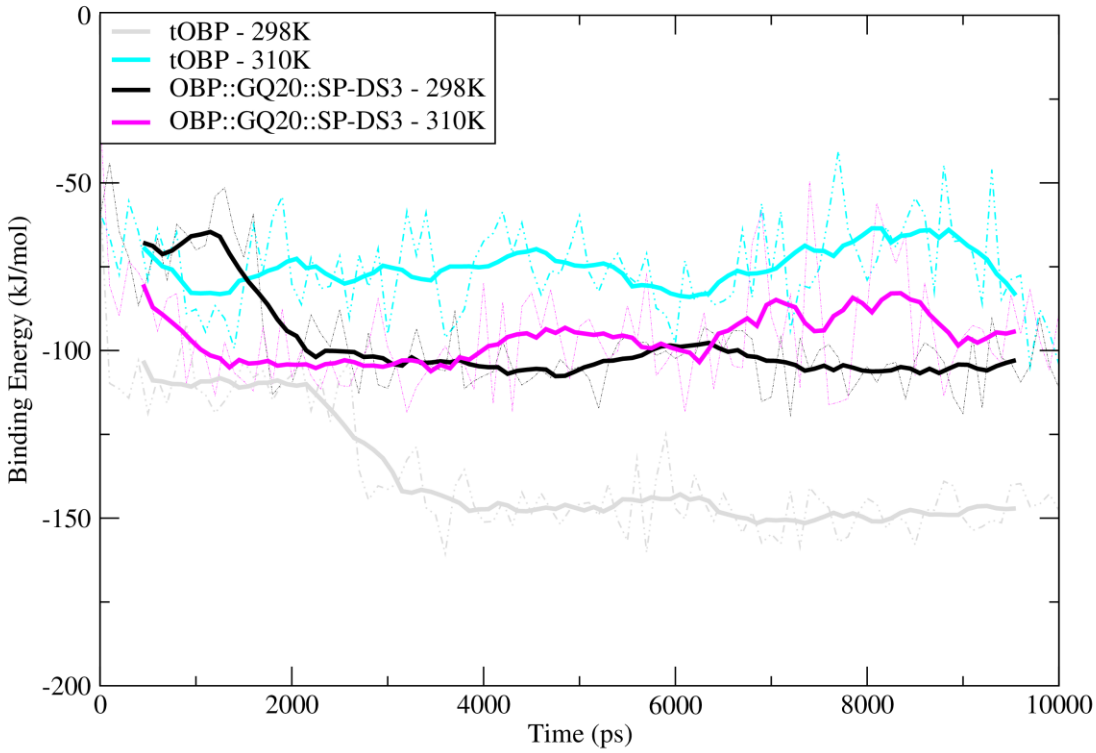

Supplementary Figure 6 | Binding energy along time obtained from g_mmpbsa tool. Running average binding curves in kJ/mol, of 1-AMA::tOBP complexes in grey and cyan, at 25 ºC and 37 ºC respectively and for 1-AMA::OBP::GQ$_{20}$::SP-DS3 complexes in black and magenta, at 25 ºC and 37 ºC respectively.
